# A Case Report of Rare Sacral Solitary Fibrous Tumor

**DOI:** 10.7759/cureus.27524

**Published:** 2022-07-31

**Authors:** Lukas Kvaščevičius, Eligijus Poškus, Donatas Petroška, Dimitrij Šeinin, Robertas Kvaščevičius

**Affiliations:** 1 Faculty of Medicine, Vilnius University, Vilnius, LTU; 2 Center for Abdominal Surgery, Vilnius University Hospital Santaros Klinikos, Vilnius, LTU; 3 National Center for Pathology, Vilnius University Hospital Santaros Klinikos, Vilnius, LTU; 4 Center for Neurosurgery, Vilnius University Hospital Santaros Klinikos, Vilnius, LTU

**Keywords:** lumbosacroiliac fusion, adjuvant radiotherapy, endovascular embolization, sacrectomy, presacral space, sacral tumors, solitary fibrous tumor, hemangiopericytoma

## Abstract

Huge primary epidural solitary fibrous tumors in the sacrum are a rare clinical entity. The purpose of this article is to present our experience in treating such large and complex neoplasms in a 31-year-old woman. The patient complained of constant nocturnal bilateral hip and lower back pain and unilateral radicular symptoms (numbness, paresthesias) in the left S1/S2 dermatomal distribution. Diagnostic imaging, biopsy, preoperative endovascular embolization, two-staged tumor resection, and lumbosacroiliac fusion were performed. The treatment resolved the patient’s neurological symptoms and resulted in overall good postoperative functionality. The patient has been in remission for more than five years despite her refusal of adjuvant radiotherapy.

## Introduction

Solitary fibrous tumor (SFT), also previously known as hemangiopericytoma, is a rare mesenchymal neoplasm of fibroblastic origin, accounting for less than 2% of all soft tissue tumors [1]. The 2013 edition of World Health Organization (WHO) Classification of Tumors of Soft Tissue and Bone [2] and the updated 2016 edition, Classification of Tumors of the Central Nervous System, introduced a new combined entity of soft tissue and meningeal hemangiopericytomas and SFTs [3], but WHO in 2021 edited this entity in the Classification of Tumors of the Central Nervous System, leaving only the term of SFT [4]. CNS SFT can be classified/graded into three grades by the presence of necroses and amount of mitoses: CNS WHO grade 1, < 2.5 mitoses/mm2 (<5 mitoses/10 high power fields [HPF]); CNS WHO grade 2, ≥ 2.5 mitoses/mm2 (≥5 mitoses/10 HPF) without necrosis; and CNS WHO grade 3, ≥ 2.5 mitoses/mm2 (≥5 mitoses/10 HPF) with necrosis [4]. Most commonly SFTs occur in the pleura and less often in extrapleural locations, such as the abdomen, pelvis, and retroperitoneal space. They can seldom be found in soft tissues of extremities, head and neck, and central nervous system. Adults between 50 and 70 years of age with no gender predilection are usually affected [1]. Several large studies in the literature had analyzed and tried to predict the local recurrence and metastasis of SFTs. In a large European multicentric cohort study, local and metastatic recurrence had occurred in 20 (12.3%) and 27 (16.7%) out of 162 patients, respectively. The calculated local recurrence incidence rates at 10 and 20 years were 19.2% and 38.6%, respectively. The metastatic recurrence incidence rates at 10 and 20 years were 31.4% and 49.8%, respectively [5]. Demicco et al. proposed the risk assessment score for predicting the development of metastasis in SFTs, which included the patient age, tumor size, mitotic activity, and the presence of necrosis. According to this study, low-risk lesions had 0% risk of metastasis at 10 years, intermediate-risk cases had 10% risk of metastasis at 10 years, and high-risk cases had 73% risk of metastasis at 5 years [6]. Modified Demicco et al.’s risk stratification system was applied to our patient. This article presents a rare primary epidural SFT at the L5/S3 levels, immunohistochemical results, and treatment strategy.

## Case presentation

**Figure 1:**
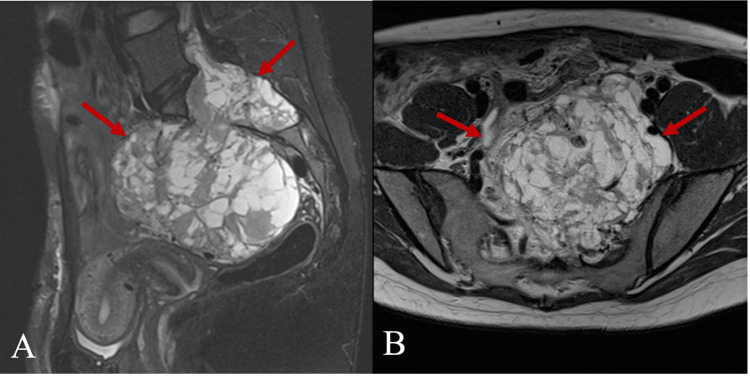
T2-weighted sagittal fat-saturated (A) and axial (B) MRI images show a huge sacral and presacral, well-defined, heterogeneous, and intermediate-to-high signal intensity tumor (arrows). Dislocation of adjacent structures (rectum, uterus, and vessels) is also visible. MRI, magnetic resonance imaging

**Figure 2:**
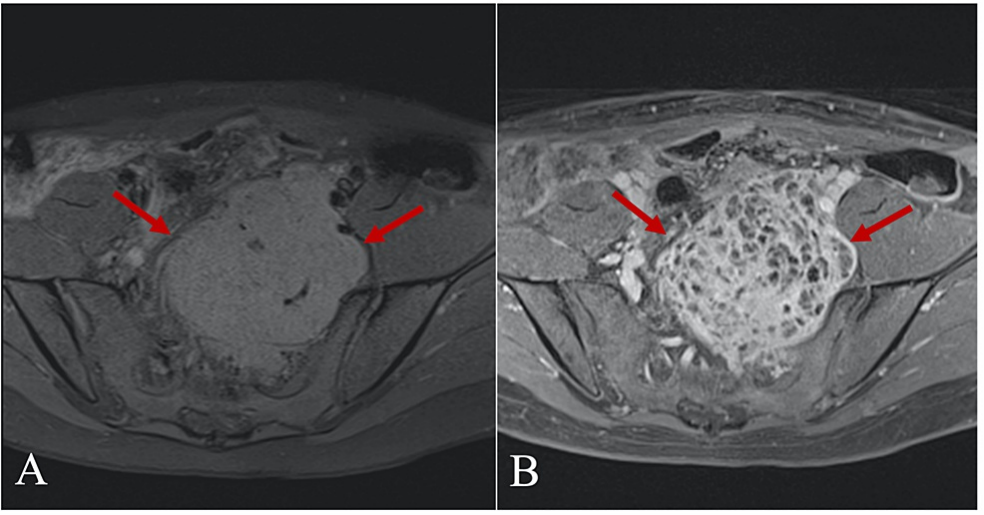
T1-weighted fat-saturated axial (A) and contrast-enhanced axial (B) MRI images. Huge sacral and presacral heterogeneous intermediate signal intensity tumor showing an avid post-contrast enhancement (arrows). No signs of infiltration to adjacent soft tissues can be seen. MRI, magnetic resonance imaging

**Figure 3:**
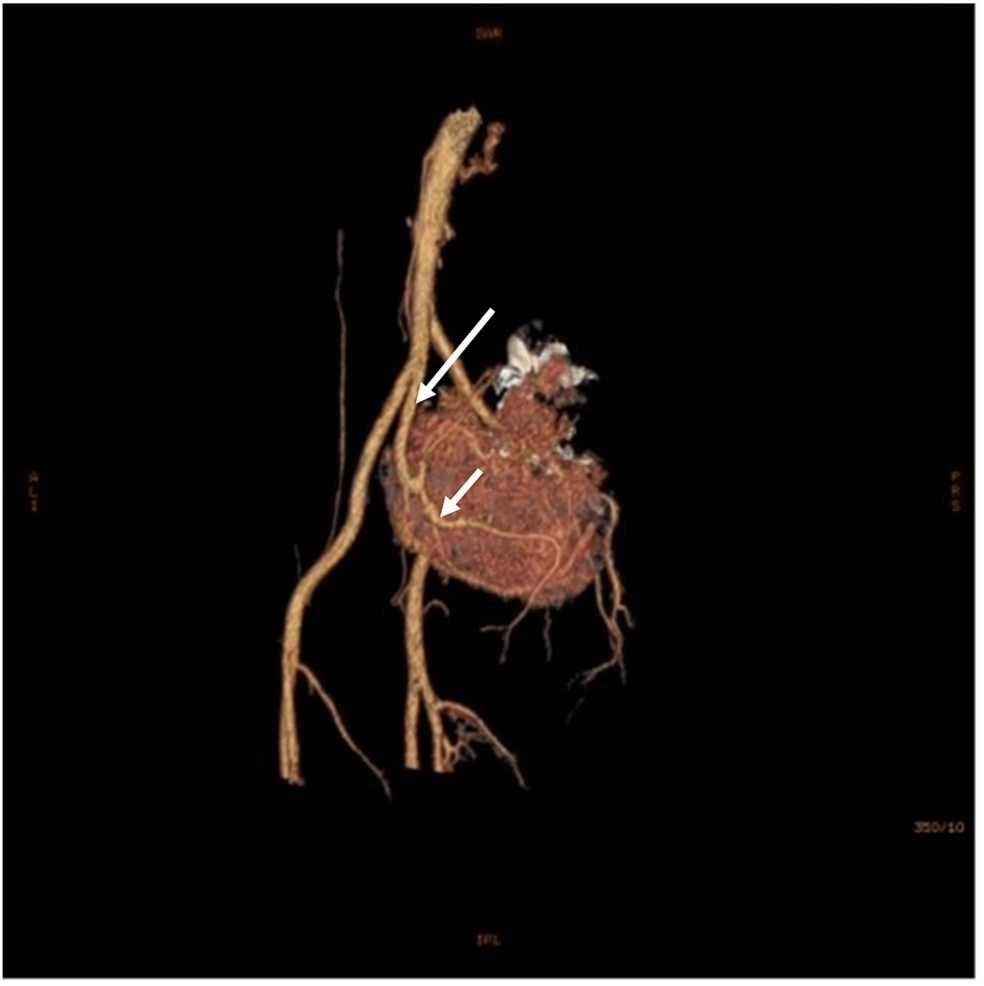
A 3D reconstruction of the MR angiography of the tumor showing its hypervascularity and a large blood-supplying vessel from the internal iliac artery (arrows). 3D, three-dimensional; MR, magnetic resonance

**Figure 4:**
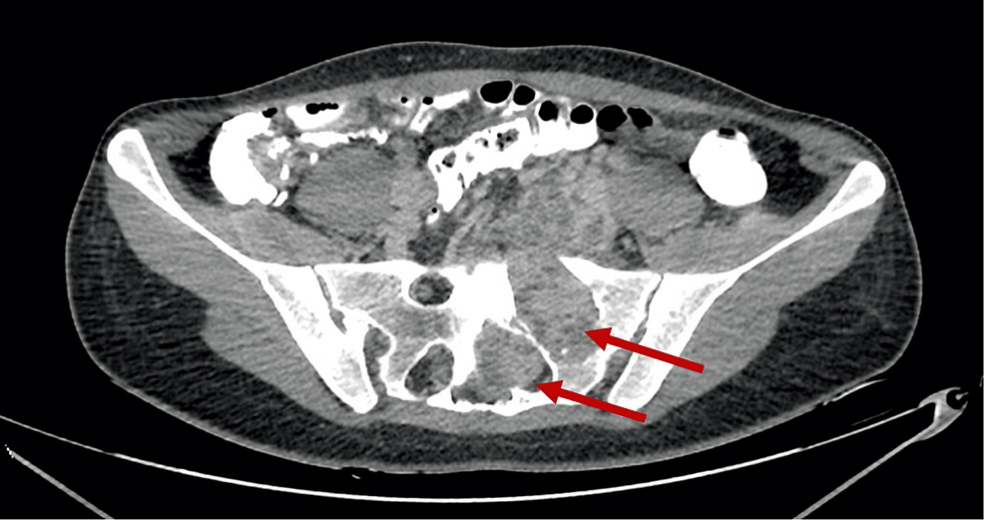
CT of the pelvis (axial view). The arrows indicate the intrasacral tumor obturating the spinal canal and sacral foramina. CT, computed tomography

A 31-year-old woman presented to the Department of Obstetrics and Gynecology in Vilnius University Hospital Santaros Klinikos for an elective laparoscopical diagnostic procedure due to a suspected ovarian cyst. She also complained of constant nocturnal bilateral hip and lower back pain and unilateral radicular symptoms (numbness, paresthesias) in the left S1/S2 dermatomal distribution that worsened over the last two years. No gynecological abnormalities were found, only a large retroperitoneal mass. Then she was referred to the Department of Neurosurgery in the same hospital where one of the authors gave further plan. Initial differentials involved more common masses in the sacral area, such as chordoma, chondrosarcoma, and giant cell tumor. There was no history of weight or appetite changes. Pelvic magnetic resonance imaging (MRI) was performed, which demonstrated a heterogeneously enhancing, multi-cystic, well-circumscribed tumor with pseudocapsule at the L5/S3 levels (Figure 1). Huge sacral tumor extension into the lumbar and sacral canal and the paravertebral area was observed, with a large portion of the tumor dislocating the uterus and rectum in the presacral area. The tumor size was 12.0 x 10.0 x 9.3 cm (Figure 1). Contrast-enhanced T1-weighted MRI images (Figure 2) and MR angiography (Figure 3) showed a hypervascular tumor with contrast enhancement in solid portions and the main blood-supplying vessel originating from the left internal iliac artery. A pelvic computed tomography (CT) scan demonstrated a large sacral tumor obturating the sacral canal and left foramina (Figure 4).

**Figure 5:**
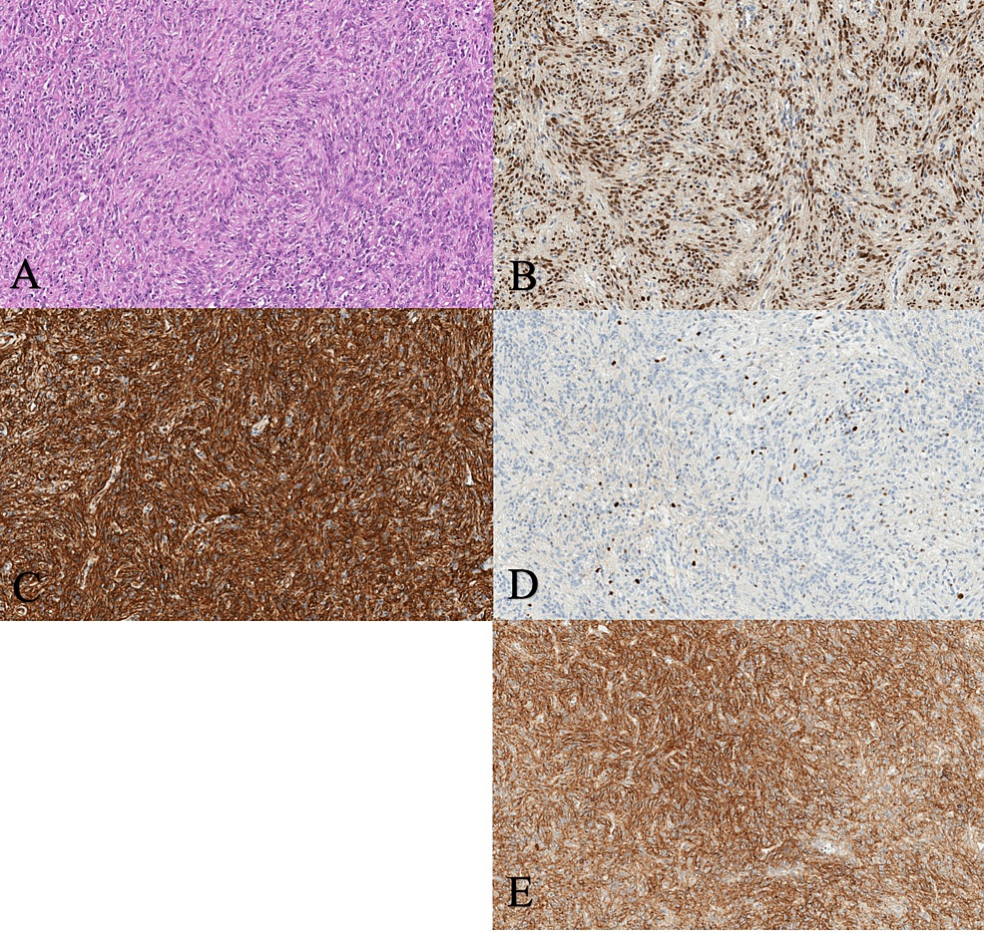
Hematoxylin and eosin staining of tumor tissue at 200x magnification shows capillaries and spindle-shaped cells with moderately atypical nuclei arranged in large palisades (A), strong diffusely positive nuclear STAT6 (B), CD34 (C), membranous (100%) and Ki67 (D) nuclear (4%) staining, and moderate CD99 cytoplasmic and membranous (80%) staining (E). STAT6, signal transducer and activator of transcription 6; CD34, cluster of differentiation 34; CD99, cluster of differentiation 99; Ki67, marker of proliferation Ki67

A fine needle biopsy was performed for histological and immunohistochemical examination. Molecular testing for *NAB2-STAT6* fusion was unavailable in our laboratory. The tumor fragments consisted of spindle-shaped cells with moderately atypical nuclei arranged in large cellular fascicles or palisades. Capillaries were neatly arranged, and no foci of necrosis or hemorrhage were observed. The mitotic index was <2 per 10 HPF. This information was of prognostic value when using the modified Demicco et al. system for predicting the risk of metastasis for the patient. Considering the age of the patient (31 years), the size of the tumor (12.0 x 10.0 x 9.3 cm), mitotic activity (<2/HPF), and the absence of necrosis in the histological view, this case should be regarded as a low-risk lesion with no risk of metastasis at 10 years. Immunohistochemical examination revealed a strong diffuse STAT6 and CD34 expression, moderately intense CD99, and weak alpha-smooth muscle actin staining; Ki67 proliferative index was around 4%. Epithelial membrane antigen (EMA) and S-100 protein were negative (Figure 5).

Surgical treatment

In the Department of Interventional Radiology, Vilnius University Hospital Santaros Klinikos, the tumor’s selective endovascular embolization was performed before surgery to reduce the risk of life-threatening intraoperative bleeding, which was followed by the two-staged surgical removal of the tumor. Using the abdominal laparotomic approach, partial resection was performed and a large portion of the tumor was removed from the presacral area with minimal blood loss. Tumor masses were separated and removed from the sacrum, dura mater, and sacral roots up to the S1/S3 level with macroscopically clear resection margins. After two weeks, through posterior partial sacrectomy, intrasacral, extradural, and vascular components of the tumor were resected, which was followed by L4-L5-S1-Ala fusion. The sacral bone defect was filled with autograft from both posterior superior iliac spines and bone substitute. The treatment improved neurological symptoms and preserved normal pelvic organ functions (defecation, urination) and sexual life. The patient was discharged after surgical interventions, and further treatment was discussed with oncologists from the same hospital. She consciously declined the suggested option of adjuvant radiotherapy due to a desire to maintain her future childbearing potential. After a year and a half, she presented to the Department of Neurosurgery in Vilnius University Hospital Santaros Klinikos with renewed lumbago and pain in her left leg during movement. The spinal X-ray and pelvic CT confirmed broken right rod at the L5/S1 level. Revision was performed and both rods were replaced. Additional posterolateral bone fusion was performed using autologous spongious autografts from L4 and L5 spinous processes and bone substitute. The patient has been followed yearly for more than five years and no recurrence of the tumor was detected.

## Discussion

Sacral SFTs are indolently growing and usually asymptomatic in early stages. However, they may cause compression symptoms ranging from radicular symptoms, lower back pain, to paresis, and less often bowel and bladder incontinence [7]. It is important to perform a biopsy for histological and immunohistochemical evaluation, which, combined with imaging, helps diagnose these tumors. Masses in this location should be differentiated from top six histological types of primary sacral tumors, such as chordoma, chondrosarcoma, giant cell tumor, neurofibroma, schwannoma, and myeloma [8]. Typically, an SFT has oval to spindle-shaped cells clustered around capillaries and may present with patternless, branching, staghorn-like appearance, and form cellular fibers or palisades with varying amounts of collagen in the histological view [9]. Malignant tumors are linked to a worse prognosis due to their higher tendency for local progression and metastasizing [10]. A highly sensitive and specific marker for the SFT is STAT6, which is positive in almost 100% of cases; a diffuse to focal but nonspecific CD34 marker is present in up to 95% of cases; CD99, a less sensitive marker, is found around 70% of cases; EMA can be expressed in 20-30% of cases [11]. However, both STAT6 and CD34 may be lost in malignant and dedifferentiated tumors. Also, it is known that desmin, cytokeratin, and S-100 protein expression are negative for SFT [9]. In our case, the tumor presented with strongly positive STAT6 and CD34, moderately positive CD99, negative EMA and S-100 markers, mitoses <5/10 HPF, and absence of necrosis, suggesting the diagnosis of grade 1 SFT.

Surgery is a fundamental treatment, and even benign SFT tumors should be treated with wide surgical resection when possible [12]. In some cases, achieving a complete resection could be technically difficult due to an unfavorable tumor location near the critical and unresectable structures. Wang et al. [13] reported that sacral tumor piecemeal resections result in comparable tumor control and progression-free survival rate, but have a lesser impact on functional outcomes than en bloc resections. We performed a two-staged piecemeal tumor resection for a couple of reasons. Firstly, we considered the length of the operation. Two separate surgeries over the period of two weeks would be less risky and more tolerable for the patient than the single-stage operation and decrease the likelihood of perioperative complications related to a long operation. Secondly, the patient's safety was considered. As the tumor mainly had presacral and intrasacral parts, two different approaches enabled us to have better control of the rectum and vasculature anterior to the sacrum and remove tumor with caution while preserving intact nerves and pelvic organs (uterus, ovaries, salpinges, bladder, ureters, bowels) and reducing blood loss to a minimum. A multicentric retrospective study suggested that combining surgical treatment with adjuvant radiotherapy reduces the risk of local recurrence, especially for partially resected or malignant extrameningeal SFT; however, its effectiveness for overall survival remains debatable [14]. In this particular case, the patient refused adjuvant radiotherapy due to the desire to save her fertility. A recent retrospective cohort study noted that a combination of surgery and chemotherapy resulted in lower overall and cancer-specific survival compared to surgery alone [15]. Still poorly understood clinical behavior and late relapse of malignancy cases suggest that a long patient follow-up is necessary [16]. After more than five years of regular yearly MRIs, there was no tumor recurrence or progression for our patient.

## Conclusions

This report described a unique case of huge primary epidural SFT in the sacrum. Before surgical removal, endovascular tumor embolization should be considered as these tumors are usually hypervascular. A two-staged piecemeal tumor resection through the combined anterior and posterior approach could be radical enough and safer than a single-staged surgery providing good control of the tumor and internal organs with surrounding structures. As a result, combined approach could preserve normal neurological function and good quality of life for the patient. To date, our patient has no signs of relapsed tumor despite her refusal of adjuvant radiotherapy.
